# Libman-Sacks Endocarditis With Triple Valvular Involvement

**DOI:** 10.7759/cureus.37734

**Published:** 2023-04-17

**Authors:** Meeran Asher Syed, Muhammad Usama Khokhar, Farhan Akbar, Muhammad Asfand, Hassan Shakoor

**Affiliations:** 1 Internal Medicine, Fauji Foundation Hospital Islamabad, Islamabad, PAK; 2 Internal Medicine, Mayo Hospital, Lahore, PAK; 3 Internal Medicine, Fauji Foundation Hospital Rawalpindi, Rawalpindi, PAK

**Keywords:** aps, sle, trivalvular, tricuspid valve endocarditis, autoimmune

## Abstract

Systemic lupus erythematosus (SLE) is a systemic inflammatory autoimmune disease with a broad spectrum of clinical manifestations. Libman-Sacks endocarditis (LSE) is due to sterile vegetations that arise in association with SLE. Nonbacterial thrombotic endocarditis, also known as marantic endocarditis, Libman-Sacks endocarditis, and verrucous endocarditis, is linked to a number of illnesses, the most prevalent of which is advanced cancer. Most often, the surfaces of mitral and aortic valves are involved. However, the involvement of the tricuspid valve is possible and is rarely described in the literature.

We present a case of a 25-year-old female who presented with LSE, lupus nephritis, and pulmonary involvement secondary to SLE. On detailed exploration, she was found to have SLE with lupus nephritis and pulmonary hypertension secondary to valvular involvement. Through this case, we would like to elaborate on the course of SLE with triple valvular involvement.

## Introduction

In the setting of systemic lupus erythematosus (SLE), Libman-Sacks (verrucous) endocarditis (LSE) is a form of non-bacterial thrombotic endocarditis (NBTE) that damages heart valves [1]. The mitral and aortic valves are the most commonly affected, while isolated tricuspid valve involvement is rare [2]. It is frequently mistaken for infectious endocarditis due to its symptomatology. Accurate diagnosis of Libman-Sacks vegetation can lead to prompt treatment and elimination of the associated complications. Close clinical and echocardiographic monitoring, as well as extensive anticoagulant or antiplatelet therapy, are recommended in SLE patients to forestall further valve degradation and thromboembolic events. Treatment of nonbacterial thrombotic endocarditis usually consists of systemic anticoagulation and therapy directed at treating the underlying malignancy or associated condition. Surgery is advised when the patient's condition deteriorates and the disease progresses [3].

## Case presentation

A 25-year-old female presented to the nephrology outpatient department with the complaint of scanty and frothy urine for 15 days. She also complained of shortness of breath and hemoptysis for 3 days. She was a known case of SLE for one year. Massive hair loss, joint pain, oral ulcer, and malar rash were the features that she had presented with a year prior. She was ANA (anti-nuclear antibodies) positive, and her anti-dsDNA (antibodies to double-stranded DNA) level was 4.10 IU/ml. Based on the points in history, examination, and investigations at that time, a diagnosis of systemic lupus erythematosus (SLE) was established.

However, she was on a regimen of hydroxychloroquine and non-steroidal anti-inflammatory drugs (NSAIDS). The disease was controlled for the duration of a year after the initial presentation. The patient did not have any worsening of symptoms during this duration, although she was non-compliant with the advice of regular follow-up.

On further exploration of her current symptoms, she revealed that her urine was scant in volume and frothy in nature. She had observed this feature 15 days prior. She developed shortness of breath (SOB) 3 days prior. It was sudden in onset, severe in intensity, and used to worsen on lying down flat. It was also associated with hemoptysis. Further, she also complained of weight gain, generalized body swelling, and sleep disturbance for the past 15 days.

In a general physical examination, the following signs were noted: In the cardiovascular system, a pan-systolic high-pitched murmur was documented on the mitral area. A pan-systolic murmur with similar features was also present on the tricuspid area. On exploration of the musculoskeletal system, joint pain, joint swelling, and joint redness were found. In mucocutaneous findings, features of hair fall and characteristic malar rash on the face were also observed. In the musculoskeletal system, small joints were found involved in a symmetrical pattern.

As the patient was a known case of SLE, a workup for infective endocarditis was initiated. However, the modified Dukes criteria was not satisfied. Blood cultures were negative. Polymerase chain reaction (PCR) was done to find out infection by HACEK organisms, but no organism from this group was found either.

A Direct Coombs test was performed which turned out to be positive. Immune hemolytic anemia along with hemoptysis explained the decreased hemoglobin levels. Details of other tests are given in Table 1:

**Table 1:** Detailed blood workup of LSE patient which includes multiple abnormal findings. s: second/s, INR: International normalized ratio, aPTT: activated partial thromboplastin time, WBC: white blood cell, RBC: red blood cell, g/dL: gram per deciliter, HCT: Hematocrit ,MCV: Mean corpuscular volume, fl: femtoliter, MCH: mean corpuscular hemoglobin, pg: picogram, MCHC: Mean corpuscular hemoglobin concentration, CPK: Creatine phosphokinase, IU/L: International units per liter, CK-MB: creatine kinase-myoglobin binding, creatine kinase-myoglobin binding: mmol/L: millimoles per liter, mm/hr: millimeters per hour.

Investigations	Results	Reference Range
Coagulation Profile		
Prothrombin Time-Control(s)	11	11-13
Prothrombin Time-Patient(s)	11	11-13
INR	1.0	~1.1
aPTT(s)	100	21-35
Hemogram		
WBC count (X10^9^/L)	4.1	4.5 - 11.0
RBC count (million cells per microliter)	2.4	3.8 - 5.2
Hemoglobin (g/dL)	5.8	11.6 - 15
HCT (%)	20	36% - 48%
MCV (fl)	93.2	80 - 100
MCH (pg)	28	27 - 31
MCHC (%)	30.1	32 - 36
Platelets (X10^9^/L)	231	150 - 400
Biochemistry		
Blood Glucose (random) (mg/dl)	112	90 to 130
Cardiac Enzymes		
CPK (IU/L)	56	25 - 200
CK-MB (IU/L)	2.20	5 to 25
Renal Function Tests		
Urea (mg/dl)	115	6 to 24
Serum Creatinine (mg/dl)	4.76	0.6 to 1.1
Serum Electrolytes		
Sodium (mmol/L)	139	136 to 145
Potassium (mmol/L)	5.47	3.6 to 5.2
Chloride (mmol/L)	113.7	96 to 106
Bicarbonate (mmol/L)	12.1	22 to 32
Inflammatory Markers		
ESR (mm/hr)	86	0 to 20
C-Reactive Protein (mg/dl)	16.5	Less than 0.3

A diagnosis of Libman-Sacks endocarditis was established. This is basically a diagnosis of exclusion. This diagnosis was supported by the above given array of test results and transesophageal echocardiography (TEE). Detailed TEE findings are given in Table 2:

**Table 2:** Detailed transesophageal echocardiography findings.

B/M Mode Dimension						Doppler	
Parameter	Result	Normal	Parameter	Result	Normal	Parameter	Result
Aortic root	33	20-40mm	LVISD	09	8-12mm	E/A	1.1
Left Atrium	38	19-39mm	LPVWD	10	7-11mm	DT	150msec
IVC	14mm	-	LVIDD	53	36-56mm	E’	12cm/sec
			LVIDS	37	25-41mm	RVSTDI	12cm/sec
			EF	57%	50-70%	AOPG	5mmHg
			FS	30%	29-37%	TVPG	61mmHg
Color Flow Mapping		MR3-4+, TR2-3+, Trivial AR				MR dp/dt	1500

This study represents the following features: Myxomatous degenerative mitral valve with severe mitral regurgitation, structurally normal other valves with aortic regurgitation, moderately severe tricuspid regurgitation, and severe pulmonary hypertension.

**Figure 1:**
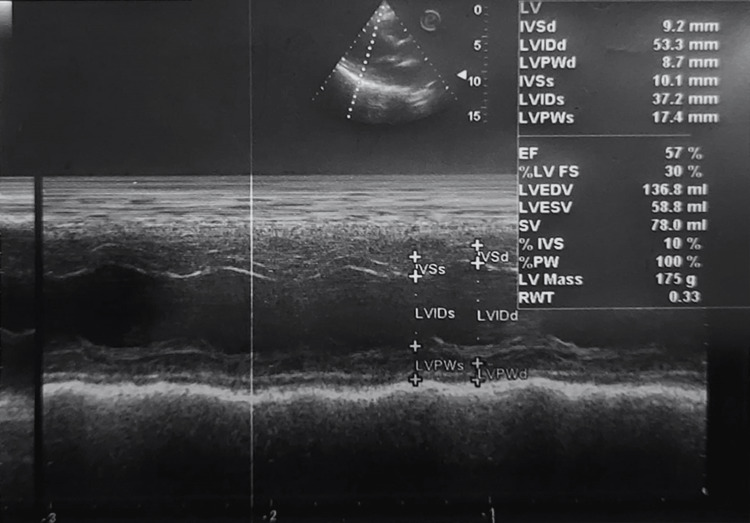
TEE representing left ventricular function and ejection fraction LV: left ventricle, IVSd: Interventricular septal end diastole, LVIDd: Left ventricular internal diameter end diastole, LVPWd: Left ventricular posterior wall end diastole, IVSs: Interventricular septal end-systole, LVIDs: Left ventricular internal diameter end-systole, LVPWs: Left ventricular posterior wall end-systole, EF: ejection fraction, LVFS: Left ventricular fractional shortening, LVEDV and LVESV: LV end-diastolic and end-systolic volumes, SV: Stroke Volume, IVS: interventricular septum, PW: Pulsed wave, RWT: Relative wall thickness

**Figure 2:**
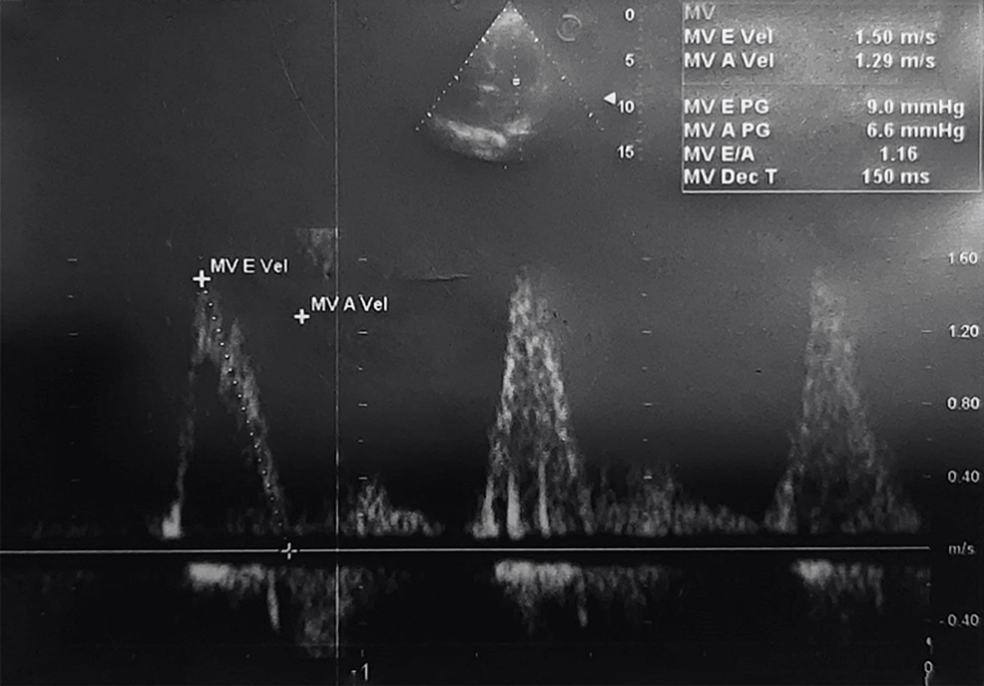
TEE representing the status of the mitral valve MV: Mitral valve, MVE Vel: mitral valve flow E wave velocity, MVA Vel: mitral valve flow A wave velocity, PG: pressure gradient, E/A: E wave/A wave ratio, MV Dec T: MV Deceleration Time

**Figure 3:**
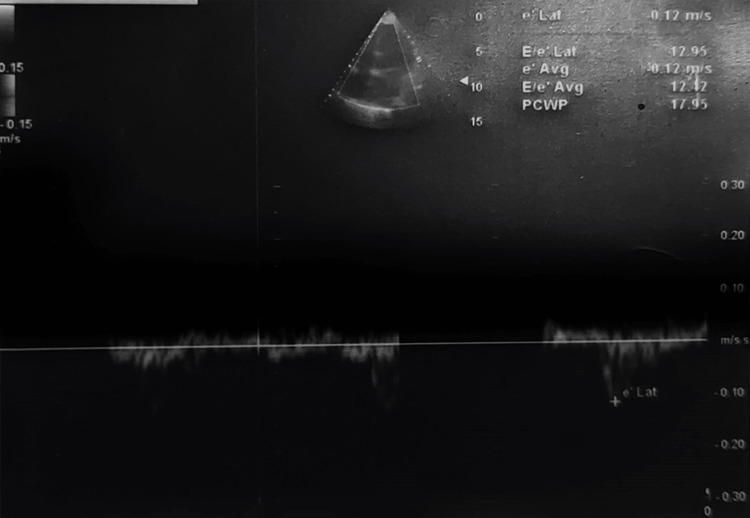
TEE assessment of pulmonary capillary wedge pressure e’lat:  diastolic left ventricular wall velocity of mitral annulus, E/e’lat: ratio of early diastolic mitral annulus velocity to early diastolic mitral annulus velocity, avg: average, PCWP: Pulmonary capillary wedge pressure

TEE showed left ventricular function and ejection fraction (Figure 1), the status of the bicuspid valve (Figure 2), and an assessment of pulmonary capillary wedge pressure (Figure 3).

Tricuspid regurgitation was primary in the given case because there was no history or evidence of Epstein’s anomaly, rheumatic heart disease, intravenous (IV) drug abuse, or right ventricular dilatation.

Features of dyspnea, exertional syncope, and exertional chest pain were exaggerated during these studies. Moreover, signs of raised jugular venous pulsation (JVP), and the prominent pulmonary component of the second heart sound were also observable. All these features of pulmonary hypertension were secondary to valvular involvement, and mitral and tricuspid regurgitation. This clinical picture correlated well with the diagnostic procedure TEE and its findings.

For supportive measures, oxygen saturation was maintained at 90-92%. Moreover, diuretics and anticoagulant with warfarin were used. Calcium channel blockers and IV prostacyclin were initiated for vasodilator function. For renal signs and symptoms, lupus nephritis was being considered. For advanced renal dysfunction following tests were performed (Table 3).

**Table 3:** Detailed results of urine examination. mg/dl: milligrams per deciliter, hpf: high power field, lpf: low power field, already mentioned abbreviations in table 1 legend are not given here.

Urine Complete Examination		
Investigations	Results	Reference Range
Physical Examination		
Color	Yellow	clear to pale yellow
Appearance	Turbid	Clear or cloudy
Chemical Examination		
Glucose	Negative	Negative
Bilirubin	Negative	Negative
Ketone	Negative	Negative
Specific Gravity	1.010	1.005 to 1.030
pH	5.5	4.6 to 8.0
Protein	++	<150 mg/dl
Urobilinogen (mg/dl)	0.57	0.5-1
Nitrite	Negative	Negative
Leukocyte esterase	Present	Negative
Microscopic Examination		
WBCs	Numerous	2-5 WBCs/hpf
RBCs	20-25	<3 RBCs/hpf
Epithelial Cells	2-3	<15-20 squamous epithelial cells/hpf
Granular Casts	Occasional	0-5 hyaline casts/lpf
Bacteria	+	None
Crystals	Negative	Occasionally

Protein/Creatinine Ratio	5.2	less than 0.2

The correlation of the clinical picture with laboratory findings pointed towards lupus nephritis. This is not uncommon in a patient of SLE. To confirm the class of lupus nephritis according to WHO classification, a renal biopsy was planned. To proceed with renal biopsy, the prerequisites included building up of blood hemoglobin levels and adjustment of CRP.

However, the clinical condition of the patient deteriorated quickly. Signs of right and left heart failure which included generalized body edema and dyspnea, palpitations, orthopnea, and paroxysmal nocturnal dyspnea flared up. The clinical picture showed doomed trends even after aggressive treatment. The intensive care unit team was involved but the patient’s vitals crashed and she expired despite intensive intervention. Left and right heart failure were the underlying cause of this clinical outcome.

## Discussion

Libman-Sacks endocarditis is a cardiac manifestation of systemic lupus erythematosus (SLE), which was first identified in 1924. SLE and antiphospholipid syndrome (APS) have both been linked to valvular involvement [4]. In as many as 61% of SLE patients who underwent transesophageal echocardiography, valvular abnormalities were found [1]. On average, pericarditis, myocarditis, LSE, pulmonary arterial hypertension, conduction dysfunction, and coronary artery disease are thought to be present in more than 50% of SLE patients. Heart valves on the left side are by far the most compromised. Although it is highly unusual, it has previously been documented that cardiac involvement is the primary cause of this presentation [5].

With a prevalence of between 0.9% and 1.6%, Libman-Sacks endocarditis is an uncommon disease that is typically discovered postmortem. LS endocarditis can affect people of any age, however, it most frequently strikes those between the ages of 40 and 80. Studies don't indicate any sex preference [6]. There are less than 10 cases reported in the literature resembling the aforementioned clinical scenario. Most probably, this is the first case being reported from Pakistan regarding trivavular involvement due to LSE/SLE to the best of our knowledge.

The patient may exhibit symptoms of heart failure driven by valve regurgitation and thromboembolic events and manifest as infective endocarditis [7]. Other important diseases to be differentiated are rheumatic valvular disease, atrial myxoma, and degenerative valvular disease.

The pathogenesis of valve lesions includes the development of fibrin-platelet thrombi on the atrophic valve. The alleged initial insult that causes the development of fibrin and platelets is endothelial damage on the valve surface. Further development of such lesions, immunoglobulin deposition, including anticardiolipin antibody and complement components, and finally distortion and valvular failure, culminate in cusp fibrosis, thickening, and scarring. The left-sided heart valves exhibit more severe blood flow turbulence and jet effects [3]. 

It is challenging to confirm the LSE diagnosis using laboratory tests. However, patients who are thought to have the condition must undergo a complete blood count, comprehensive metabolic workup, blood cultures, autoimmune profile, and hypercoagulable workup [5]. Three-dimensional (3D) transesophageal echocardiography (TEE) may provide improved identification, characterization, and clinical correlations of Libman-Sacks vegetations despite the excellent diagnostic value of two-dimensional (2D) TEE for Libman-Sacks vegetation detection [8].

In order to lessen the inflammatory response brought on by LSE, corticosteroids are thought of as a possible treatment; nevertheless, they can result in tissue scarring and fibrosis, predisposing to further valvular damage. In SLE patients, especially those who have had prior thromboembolic episodes, anticoagulation should be taken into consideration as secondary prophylaxis for thromboembolic events. For cases of LSE with symptoms and severe disease, surgical valve replacement is advised [5].

## Conclusions

Libman-Sacks endocarditis is a rare but very characteristic manifestation of SLE. The involvement of the mitral and aortic valves is fairly described in the literature; however, the involvement of the tricuspid valve is rare in nature. We deem this case report crucial addition to medical literature because it describes the aggressive nature of LSE, especially with tricuspid valve involvement. Moreover, a vigorous clinical picture when LSE presents with a combination of pulmonary hypertension and lupus nephritis, emphasizes the importance of intensive care in such patients. However, our patient expired despite of aggressive measures due to right and left heart failure. This case can also help in redefining biostatistics regarding valvular involvement and mortality associated with it.

## References

[1] Keenan JB, Rajab TK, Janardhanan R, Larsen BT, Khalpey Z (2016). Aortic valve replacement for Libman-Sacks endocarditis. BMJ Case Rep.

[2] Wang Y, Ma C, Yang J (2015). Libman-sacks endocarditis exclusively involving the tricuspid valve in a patient with systemic lupus erythematosus. J Clin Ultrasound.

[3] Unic D, Planinc M, Baric D, Rudez I, Blazekovic R, Senjug P, Sutlic Z (2017). Isolated tricuspid valve Libman-Sacks endocarditis in systemic lupus erythematosus with secondary antiphospholipid syndrome. Tex Heart Inst J.

[4] Akhlaq A, Ali TA, Fatimi SH (2016). Mitral valve replacement in systemic lupus erythematosus associated Libman-Sacks endocarditis. J Saudi Heart Assoc.

[5] Al-Jehani M, Al-Husayni F, Almaqati A, Shahbaz J, Albugami S, Alameen W (2021). A case of systemic lupus erythematosus in a patient presenting with Libman-Sacks endocarditis. Case Rep Cardiol.

[6] Ibrahim AM, Siddique MS (2022). Libman Sacks endocarditis. In. https://www.ncbi.nlm.nih.gov/books/NBK532864/.

[7] Ruiz D, Oates JC, Kamen DL (2018). Antiphospholipid antibodies and heart valve disease in systemic lupus erythematosus. Am J Med Sci.

[8] Roldan CA, Tolstrup K, Macias L, Qualls CR, Maynard D, Charlton G, Sibbitt WL Jr (2015). Libman-Sacks endocarditis: detection, characterization, and clinical correlates by three-dimensional transesophageal echocardiography. J Am Soc Echocardiogr.

